# Negative Affectivity, Depression, and Resting Heart Rate Variability (HRV) as Possible Moderators of Endogenous Pain Modulation in Functional Somatic Syndromes

**DOI:** 10.3389/fpsyg.2018.00275

**Published:** 2018-03-06

**Authors:** Maaike Van Den Houte, Lukas Van Oudenhove, Ilse Van Diest, Katleen Bogaerts, Philippe Persoons, Jozef De Bie, Omer Van den Bergh

**Affiliations:** ^1^Health Psychology, Faculty of Psychology and Educational Sciences, University of Leuven, Leuven, Belgium; ^2^REVAL – Rehabilitation Research Center, Faculty of Medicine and Life Sciences, Hasselt University, Diepenbeek, Belgium; ^3^Department of Psychiatry, University Hospital Gasthuisberg, Leuven, Belgium; ^4^Laboratory for Brain-Gut Axis Studies, Translational Research Center for Gastrointestinal Disorders, University of Leuven, Leuven, Belgium; ^5^Centre for Translational Psychological Research, Hospital ZOL Limburg, Genk, Belgium

**Keywords:** endogenous pain modulation, conditioned pain modulation, counter-irritation, fibromyalgia, chronic fatigue syndrome, heart rate variability, negative affectivity, depression

## Abstract

**Background:** Several studies have shown that patients with functional somatic syndromes (FSS) have, on average, deficient endogenous pain modulation (EPM), as well as elevated levels of negative affectivity (NA) and high comorbidity with depression and reduced resting heart rate variability (HRV) compared to healthy controls (HC). The goals of this study were (1) to replicate these findings and (2) to investigate the moderating role of NA, depression, and resting HRV in EPM efficiency within a patient group with fibromyalgia and/or chronic fatigue syndrome (CFS). Resting HRV was quantified as the root mean square of successive differences between inter-beat intervals (RMSSD) in rest, a vagally mediated time domain measure of HRV.

**Methods:** Seventy-eight patients with fibromyalgia and/or CFS and 33 HC completed a counter-irritation paradigm as a measure of EPM efficiency. Participants rated the painfulness of electrocutaneous stimuli (of individually calibrated intensity) on the ankle before (baseline phase), during (counter-irritation phase) and after (recovery phase) the application of a cold pain stimulus on the forearm. A larger reduction in pain in the counter-irritation phase compared to the baseline phase reflects a more efficient EPM.

**Results:** In contrast to our expectations, there was no difference between pain ratings in the baseline compared to counter-irritation phase for both patients and HC. Therefore, reliable conclusions on the moderating effect of NA, depression, and RMSSD could not be made. Surprisingly, patients reported more pain in the recovery compared to the counter-irritation and baseline phase, while HC did not. This latter effect was more pronounced in patients with comorbid depression, patients who rated the painfulness of the counter-irritation stimulus as high and patients who rated the painfulness of the electrocutaneous stimuli as low. We did not manage to successfully replicate the counter-irritation effect in HC or FSS patients. Therefore, no valid conclusions on the association between RMSSD, depression, NA and EPM efficiency can be drawn from this study. Possible reasons for the lack of the counter-irritation effect are discussed.

## Introduction

Endogenous pain modulation refers to the internal modification of pain signals in order to accommodate the body’s current and future needs (Bourne et al., 2014). One example of EPM is DNIC, where neural responding to noxious stimulation in one area of the body is reduced by the application of noxious stimulation in another area of the body (“pain inhibits pain”; Le Bars et al., 1979). In humans, this mechanism is tested and quantified with counter-irritation paradigms, also referred to as conditioned pain modulation paradigms. In these paradigms, pain elicited by a single stimulus (the test stimulus) is compared with pain elicited by the same stimulus when accompanied by a painful stimulus of another modality (the counter-irritation stimulus) in a different area (“pain inhibits pain”). EPM efficiency is then defined as the difference in the physiological or subjective pain response to the test stimulus before versus during the application of the counter-irritation stimulus, with a larger reduction in painfulness of the test stimulus during counter-irritation reflecting more efficient EPM. Studies using these paradigms have established that on average EPM is less efficient in patients with chronic pain conditions (Lewis et al., 2012; Staud, 2012; Yarnitsky, 2015), and particularly in patients with FSS, like irritable bowel syndrome (Wilder-Smith, 2011), fibromyalgia (Julien et al., 2005; Jensen et al., 2009), and CFS (Meeus et al., 2008). Moreover, prospective studies have shown that EPM efficiency before surgery is predictive for the development of post-operative chronic pain (Yarnitsky et al., 2008; Wilder-Smith et al., 2010). Although the exact mechanisms behind these findings need further exploration, it has been suggested that the dynamic interplay between descending facilitatory and inhibitory components, as measured by counter-irritation tasks, plays a significant role in the spread of central sensitization (Wilder-Smith et al., 2010), which has been put forward as an important neurophysiological process underlying FSS (Bourke et al., 2015). Because of its predictive value, it is thought that the performance on counter-irritation paradigms reflects individual differences in the susceptibility to develop chronic pain conditions, and assessment of EPM efficiency might help prevent the development of chronic pain by early identification of patients at risk (Edwards, 2005). Furthermore, several pharmacological (e.g., opioids, serotonergic drugs) and non-pharmacological (e.g., cognitive-behavioral therapy) interventions can target reduced EPM efficiency and consequently contribute in alleviating chronic pain in patients with low EPM efficiency (Wilder-Smith, 2011).

Functional somatic syndromes are disorders characterized by chronic and debilitating symptoms that are not sufficiently explained by an organic, identifiable disease. Although on average EPM efficiency seems to be reduced in FSS patients, EPM efficiency within the FSS population is heterogeneous (Lautenbacher and Rollman, 1997; Staud et al., 2003; Potvin and Marchand, 2016). Presumably this is due to the large number of factors influencing EPM efficiency, including methodological factors (such as the intensity of the counter-irritation stimulus), physiological factors and psychological factors and their interactions (Pud et al., 2009; Nahman-Averbuch et al., 2015, 2016; Van Den Houte et al., 2017b). For instance, it has been shown that high levels of anxiety, depression, and pain catastrophizing might interfere with efficient EPM, but that the strength of these relationships might depend on stimulus modality.

Fibromyalgia and CFS are complex disorders, and the interaction between multiple biological, psychological, and social factors influence the onset and course of the symptoms (Van Houdenhove and Luyten, 2008). Therefore, heterogeneity within the fibromyalgia/CFS population is high. For instance, patients may vary with regard to NA, the tendency to experience negative emotional states in daily life (Watson and Clark, 1984). NA is related to symptom reporting in the healthy population (Van Diest et al., 2005) and to functional impairment in fibromyalgia (Cosci et al., 2011). Individuals with high NA have higher risk of developing a mental (Zinbarg et al., 2016) or (functional) somatic (Smith and Mackenzie, 2006) illness. The correlation between NA and somatic symptoms in a healthy population is translated by higher prevalence of mood and anxiety disorders in patients with FSS (Henningsen et al., 2003). NA influences affective processing of pain (Harkins et al., 1989) and predicts changes in pain tolerance after vaccination (Lacourt et al., 2015). Moreover, evidence suggests an important role for the serotonergic system in EPM (Treister et al., 2011). The serotonergic system has a primarily inhibitory function, and is critically involved in modulating memory, mood, sleep, and appetite (Jacobs and Azmitia, 1992). Importantly, the serotonergic system is believed to be out of balance in depression (Naughton et al., 2000), indicating a possible link between EPM deficiency and depression. Indeed, one study using a counter-irritation paradigm found that the inhibitory effect of the counter-irritation stimulus on pain ratings for the test stimulus was reduced in fibromyalgia patients compared to controls, but that this deficit was even stronger in fibromyalgia patients with comorbid depression (de Souza et al., 2009). Therefore, the first goal of this study was to replicate these results and additionally explore the relationship between NA and EPM efficiency within a patient group with fibromyalgia and/or CFS.

Next to elevated levels of depression and NA, patients suffering from fibromyalgia and CFS have, on average, lower levels of resting HRV (Meeus et al., 2013). HRV refers to the variability in the interval between consecutive heart beats, or R–R intervals. Vagally mediated measures of HRV, such as the RMMSD, are thought to reflect parasympathetic tone, with higher variability reflecting better inhibitory control. Although multiple studies have demonstrated that patients with functional pain disorders have, on average, reduced resting HRV (Mazurak et al., 2012; Meeus et al., 2013), only a few studies have focused on the relationship between HRV and the processing of experimentally induced pain (Koenig et al., 2014). A relationship between resting HRV and EPM efficiency has been found in healthy samples (Tsao et al., 2013; Nahman-Averbuch et al., 2015; Van Den Houte et al., 2017b), but to our knowledge the relationship between EPM efficiency and resting HRV has not been studied in a FSS patient sample. Therefore, the second goal of this study was to investigate the relationship between HRV in rest and EMP efficiency within a patient group with fibromyalgia and/or CFS.

In order to investigate the relationship between EPM and depression, NA, and HRV in FSS patients, patients diagnosed with fibromyalgia and/or CFS and HC went through a standard counter-irritation paradigm. Subjective pain elicited by electrocutaneous stimuli was assessed before, during and after counter-irritation with a CPAW. We expected that pain ratings elicited by the electrocutaneous stimuli would be lower during counter-irritation compared to pain ratings before counter-irritation, but that this pain reduction would be smaller in patients compared to controls, as has been found in previous studies (Staud, 2012). Furthermore, within the patient group we expected that EPM deficiency, as defined by a smaller pain reduction effect, would be related to higher NA, the presence of depression and lower HRV.

## Materials and Methods

### Participants

Patients were recruited in the Department of Psychiatry of the Ziekenhuis Oost-Limburg (ZOL) Hospital (Genk) and University Hospital Gasthuisberg (Leuven) and through the Rheumatology Center in Genk. Only patients with a doctor-based diagnosis for fibromyalgia, CFS, or both were included. Further, participants filled out a questionnaire to check for fulfillment of the 2010 ACR criteria for fibromyalgia (Wolfe et al., 2010) and the 1994 CDC criteria for CFS (Fukuda et al., 1994). Exclusion criteria for patients were a BMI > 35, pregnancy, an electronic implant, anorexia, or bulimia nervosa, (history of) psychosis, alcohol- or drug dependence and chronic cardiovascular, respiratory or neurological disorders. Healthy controls were recruited through local advertisement and matched for age and gender through frequency sampling. In order to investigate moderators of EPM efficiency within the patient group, we recruited twice the number of patients compared to HC. An additional exclusion criterion for HC was the presence of any self-reported chronic somatic disorders or the presence of any psychiatric disorders, as determined by a neuropsychiatric diagnostic interview (see further). All participants provided written informed consent before participating in the study. Participants were asked to abstain from smoking, caffeine, and sports for 4 h prior to the test session, and from alcohol 24 h prior to the test session. The study was approved by the Medical Ethical Committees of Hospital ZOL (Genk; Approval No. 14/012L) and University Hospital Gasthuisberg (Leuven; Approval No. 14/012L S56413).

### Design

The study described in this paper is part of a larger study on symptom perception and stress reactivity in patients with fibromyalgia and CFS. Participants completed a psychiatric diagnostic interview, filled out a questionnaire battery and participated in a test session that took place either in University Hospital Gasthuisberg (Leuven) or Hospital ZOL (Campus Sint-Barbara, Lanaken). Because of the large influence of circadian rhythms on physiological measures, this test session always took place from 2 to 5 pm. The test session consisted of a non-invasive baseline measurement of physiological parameters and four well-validated paradigms. In a first paradigm, the picture viewing paradigm, participants watched a positive, negative, and neutral picture series and were asked to rate their mood and physical symptoms after every picture series. In a second paradigm, a rebreathing paradigm, dyspnea perception was investigated. Dyspnea was induced with CO_2_ inhalation by means of a rebreathing bag, gradually increasing FetCO_2_-levels, respiratory flow, and perceived dyspnea. Perceived dyspnea, respiratory flow, and FetCO_2_ levels were continuously measured during a room air baseline phase (60 s), rebreathing phase (150 s), and room air recovery phase (150 s). The third paradigm was a fear conditioning paradigm, in which an unconditioned stimulus (a picture of a fearful woman and an accompanying scream through the headphones) was paired with a conditioned danger cue (CS+) but never with a conditioned safety cue (CS-). The conditioned stimuli were circles of varying sizes. Fear-potentiated startle and US expectancy were measured during a conditioning phase and a generalization phase. The last paradigm was a counter-irritation paradigm (see further for detailed methods). Only the results of the counter-irritation paradigm are described here. A detailed description of the methods and results of the picture viewing paradigm and the rebreathing paradigm can be found elsewhere (Van Den Houte et al., 2017a, 2018).

### Measures

#### Self-Report Measures

**Psychiatric comorbidity** was assessed by the experimenter over the phone using the Dutch version of the MINI International Neuropsychiatric Interview 5.0.0., based on the DSM-IV (Sheehan et al., 1998; Overbeek et al., 1999). Fulfillment of the criteria for the following psychiatric disorders was assessed: depressive episode, hypomania, panic disorder, agoraphobia, social phobia, obsessive-compulsive disorder, post-traumatic stress disorder, alcohol dependency, drug dependency, psychotic disorder, anorexia nervosa, bulimia nervosa, generalized anxiety disorder (GAD), and somatization disorder. HC were excluded from participation if they fulfilled criteria for one of the disorders, patients were excluded from participation if they fulfilled criteria for alcohol- or drug- dependency, psychotic disorder or anorexia or bulimia nervosa. Patients’ results on the fulfillment of the criteria for “presence of a current depressive episode” were used to assess comorbid **depression** in this study.

**Negative affectivity** was measured with the Negative Affect subscale of the Positive and Negative Affect Schedule (Watson et al., 1988). Respondents answer on a 5-point Likert scale to what extent (1: not at all – 5: very much) they experience each of ten negative emotions in daily life (trait version) or at that moment (state version). Trait NA was measured before the test session in an online questionnaire battery, state negative affect was measured immediately before the counter-irritation paradigm.

**Pain intensity** for the electrocutaneous stimuli and the CPAW was measured with a vertical numeric rating scale presented on the computer screen. Labels next to the scale were no pain (0), very slight – barely noticeable (5), very slight (10), slight (20), moderate (30), rather strong (40), strong (50), very strong (60–80), very very strong (90), and unbearable (100). Participants could click inside of the scale to indicate their pain rating, and press the spacebar to confirm their chosen rating. During pain rating, the exact numeric rating the participant had chosen was always presented on the screen next to the rating scale.

##### Baseline heart rate variability

Baseline HRV was derived from a 10-min ECG recording during which the participant was instructed to sit still and relax. Three disposable 8-mm Ag/AgCl electrodes were placed underneath the participant’s right and left clavicle and at the left lower ribs. The signal was sampled at 1000 Hz and fed into a Coulbourn V75-04 Bioamplifier (Allentown, PA, United States). The ECG recording was visually inspected and processed offline with the ECG processing software ARTiiFACT (Kaufmann et al., 2011), which was also used to derive R–R intervals from which the RMSSD was calculated as a time–domain parameter. This parameter has been shown to be a good measure of vagally mediated HRV (Thayer et al., 2012).

### Noxious Stimuli

Electrocutaneous stimulation (80-ms train of 10 × 1 ms pulse) was delivered with a constant current stimulator (Digitimer DS7, Digitimer, Welwyn Garden City, England). Two 8-mm Ag/AgCl electrodes filled with K-Y jelly were attached to the right ankle. The intensity of the electrical stimulation was determined individually with a calibration procedure, during which participants received electrocutaneous stimuli of increasing intensity. Participants were asked to rate each stimulus on a scale from 0 to 10 (0: *I don’t feel the stimulus;* 1: *I’m aware of the stimulus but it is not painful, it is merely a sensation;* 2: *The stimulus is not painful yet but it is unpleasant;* 3: *The stimulus is mildly painful* up to 10: *The stimulus is unbearably painful*). The participants were encouraged to select the stimulus intensity scoring 8, meaning that the *stimulus is painful and takes some effort to tolerate, but is tolerable for a few times.* During the calibration procedure, the following intensity steps were used (until the participant indicated not wanting to go any higher): 1, 2, 4, 6, 8, 11, 14, 17, 20, 24, 28, and 32 mA.

Cold pain was delivered with a CPAW (Porcelli, 2014). Two cold gel packs (approximately 1°C; 15 × 28 cm) were attached to a piece of fabric with Velcro. The folded CPAW was wrapped entirely around the forearm of the non-dominant hand and fastened by two Velcro straps.

### Procedure

The counter-irritation paradigm used in this study is adapted from Piché et al. (2011). First, participants filled out the PANAS (state version). Before the counter-irritation paradigm, participants went through the calibration procedure, in which the electrocutaneous stimulus intensity was individually determined. After this, participants were told that they would receive electrocutaneous stimuli that might vary in intensity, but would never be higher than the intensity that was selected. In reality, participants received the chosen intensity throughout the entire counter-irritation paradigm.

Participants received 20 electrocutaneous stimuli in total during the counter-irritation paradigm. The first five stimuli were used as stabilization stimuli and were not used for analysis, stimuli 6–10 were used as baseline stimuli, stimuli 11–15 were accompanied by the CPAW and were used as the counter-irritation stimuli, and stimuli 16–20 were used as recovery stimuli since the CPAW was then removed. The first ten stimuli were delivered consecutively. Stimuli 10 and 11 and stimuli 15 and 16 were separated by a pause in which the researcher attached/removed the CPAW. All other electrocutaneous stimuli were delivered with an ITI of 12 s. Immediately after every electrocutaneous stimulus, the pain rating scale was shown on the screen and participants could click on the scale to indicate how painful the electrocutaneous stimulus was for them. The participants were told they should only rate the painfulness of the electrocutaneous stimuli. The participants could click multiple times inside the rating scale to indicate their pain rating and had to press spacebar to confirm their response. If the participants did not enter spacebar before the next stimulus was delivered, the response was not registered. After the CPAW was removed (before stimulus 16), participants were also asked to rate the painfulness of the CPAW on an equivalent rating scale.

### Planned Statistical Analyses

Average pain ratings in the baseline phase, counter-irritation phase and recovery phase were calculated for every participant.

To investigate differences in EPM efficiency between patients and HC, a mixed model analysis was performed on the average pain ratings, with phase (baseline, counter-irritation, and recovery; within-subject) and group (patient and HC; between-subject) as fixed effects. Within this analysis, we tested specific hypotheses by comparing pain ratings in the different phases with each other in patients and HC separately.

To investigate moderators of EPM efficiency within the patient group, several mixed model analyses were performed within the patient group, with phase and the moderator variable (logarithmically transformed and centered RMMSD and centered NA as continuous variables and depression as a dichotomous variable) as fixed effects in separate analyses. When appropriate, we performed follow-up tests by comparing pain ratings in the different phases with each other at different levels of the moderator.

The reported results are derived from mixed model analyses with an unstructured covariance matrix. Different more parsimonious mixed models were additionally run, with covariance structures that are conceivable given the design (auto-regressive, heterogeneous auto-regressive, and compound symmetry covariance). These latter models did not improve model fit (assessed with Akaike’s Information Criterion, AIC) compared to the models with an unstructured covariance matrix. Changing the covariance structure had very little impact on the *F*- and *p*-values, hence the results are robust for changes in variance–covariance structure.

All reported *p*-values from specific hypothesis testing and follow-up tests were corrected for multiple testing with the stepdown Bonferroni method. All analyses were performed with SAS 9.4.

## Results

### Sample Characteristics

Eighty-one patients (mean age: 42.11, *SD* = 10.62; 71 women) and 41 HC (mean age: 42.37, *SD* = 11.38; 36 women) participated in the study. Data of the counter-irritation paradigm could not be used for 7 HC and 2 patients due to technical malfunctions. Data of 1 HC and 1 patient could not be used because they misunderstood the instructions. The final sample therefore consisted of 78 patients and 33 HC. Mean levels of NA, RMSSD, and perceived pain from the CPAW for patients and HC are presented in **Table 1**. Patients had higher levels of NA, but did not differ from HC with regards to RMSSD and perceived pain from the CPAW. Thirty-six patients (46.2%) fulfilled the criteria for current depressive episode as assessed by the MINI. Patients suffering from a depression had lower levels of RMSSD compared to patients not suffering from a comorbid depression (*t*_70_ = -2.00, *p* = 0.050). Fifteen patients (19.2%) were taking non-steroidal anti-inflammatory drugs (NSAID), 26 patients (33.3%) were taking opioids and 23 patients (29.5%) were taking paracetamol at the time of the test session. Forty-two patients (53.8%) were taking at least one type of analgesic, 36 patients (46.2%) were taking at least one type of antidepressant. Patients taking antidepressants had lower levels of RMSSD compared to patients who were not taking antidepressants (*t*_69_ = 2.10, *p* = 0.040), but RMSSD was not related to the use of analgesics. The intensity of the electrocutaneous stimuli used in the counter-irritation paradigm was determined individually during a calibration procedure. The median chosen intensity was, for both groups, 11 mA. An exact Wilcoxon rank sum test indicated that the distribution of the used intensity was equal in both groups (*Z* = 1.42, *p* = 0.16).

**Table 1:** Means and standards deviations for patients and HC for NA, RMSSD, cold pain, and age.

	Patients		HC		*t*	*df*	*p*
	Mean	*SD*	Mean	*SD*			
NA (trait)	27.45	8.98	15.79	4.36	9.15	106.28	<0.001
Negative affect (state)	13.76	5.21	11.38	3.01	3.00	95.65	0.003
RMSSD	33.21	24.34	32.70	14.52	0.13	90.30	0.90
Cold pain	35.35	28.81	29.71	28.53	0.91	98	0.37
Age	42.28	10.78	40.52	10.87	-0.78	109	0.43

*HC, healthy controls; NA, negative affectivity; RMSSD, root mean square of successive differences of R–R intervals*.

### Hypothesis Testing

#### EPM Efficiency in Patients and HC

**FIGURE 1:**
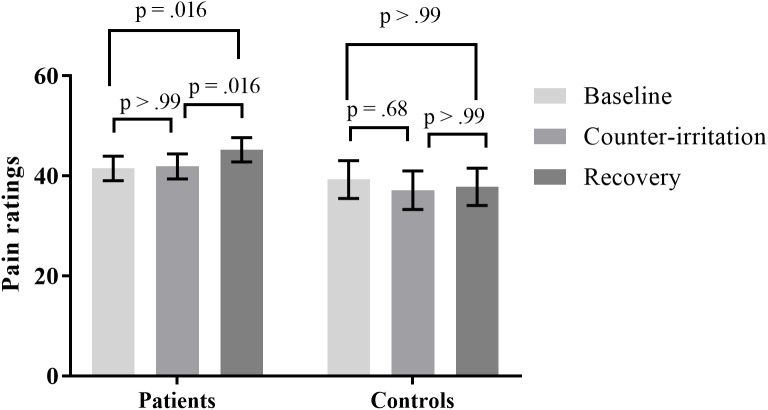
Least square mean pain ratings for electrocutaneous stimuli for patients and controls in the baseline, counter-irritation and recovery phase. *p*-values are corrected for multiple testing with the stepdown Bonferroni method. Vertical bars denote standard errors.

Pain ratings during the counter-irritation paradigm overall did not differ by phase (main effect of phase: *F*_2,109_ = 2.08, *p* = 0.13) nor by group (main effect of group: *F*_1,109_ = 1.21, *p* = 0.27). There was a trend for a group × phase interaction effect (*F*_2,109_ = 2.76, *p* = 0.068). The specific hypothesis test indicated that pain ratings in the baseline phase did not differ from pain ratings in the counter-irritation phase for patients (*t*_109_ = 0.37, *p* > 0.99, Cohen’s *d* = 0.02) or HC (*t*_109_ = -1.38, *p* = 0.68, Cohen’s *d* = 0.10). Pain ratings in the counter-irritation phase were significantly lower than pain ratings in the recovery phase for patients (*t*_109_ = 3.08, *p* = 0.016, Cohen’s *d* = 0.15), but did not differ from each other for HC (*t*_109_ = 0.41, *p* > 0.99, Cohen’s *d* = 0.03). Pain ratings in the recovery phase were higher than pain ratings in the baseline phase for patients (*t*_109_ = 3.08, *p* = 0.016, Cohen’s *d* = 0.17), but did not differ from each other in HC (*t*_109_ = -0.79, *p* > 0.99, Cohen’s *d* = 0.07), see **Figure 1**.

#### Effect of NA Within Patients

When controlling for NA, the effect of phase on pain ratings was not significant (main effect of phase: *F*_2,75_ = 0.22, *p* = 0.80). Patients scoring higher on NA gave higher pain ratings overall (main effect of NA: *F*_1,75_ = 4.24, *p* = 0.043), but this did not differ between phases (NA × phase interaction effect: *F*_2,75_ = 0.56, *p* = 0.57). Therefore, follow-up analyses were not performed.

#### Effect of Depression Within Patients

**FIGURE 2:**
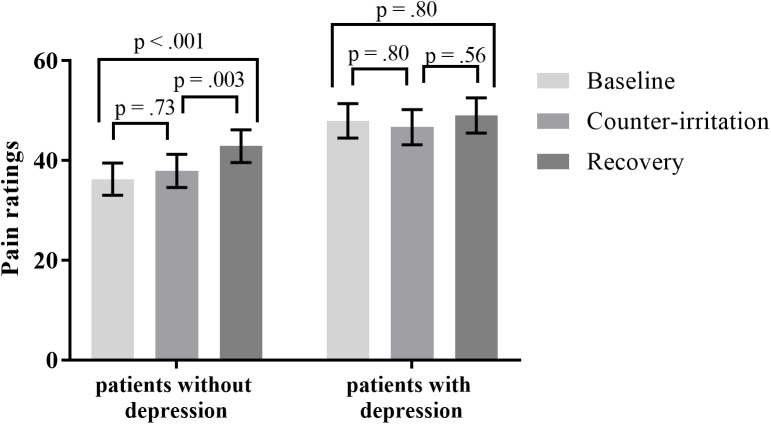
Least square mean pain ratings for electrocutaneous stimuli for patients with and without comorbid depression, in the baseline, counter-irritation, and recovery phase. Depression was diagnosed by the MINI International Neuropsychiatric Interview 5.0.0. *p*-values are corrected for multiple testing with the stepdown Bonferroni method. Vertical bars denote standard errors.

When controlling for depression, the effect of phase on pain ratings was significant (main effect of phase: *F*_2,75_ = 6.80, *p* = 0.002). There was a trend for a main effect of depression on pain ratings during the counter-irritation paradigm (*F*_1,75_ = 3.68, *p* = 0.059) and a trend for a depression × phase interaction effect (*F*_2,75_ = 2.65, *p* = 0.077). Follow-up analyses indicated that electrocutaneous pain ratings in the baseline phase did not differ from electrocutaneous pain ratings in the counter-irritation phase for patients without (*t*_75_ = 1.18, *p* = 0.73, Cohen’s *d* = 0.08) or with (*t*_75_ = -0.85, *p* = 0.80, Cohen’s *d* = 0.06) a comorbid depression. Patients without comorbid depression had higher pain ratings in the recovery phase compared to the baseline phase (*t*_75_ = 4.01, *p* < 0.001, Cohen’s *d* = 0.31) and the counter-irritation phase (*t*_75_ = 3.37, *p* = 0.006, Cohen’s *d* = 0.23), while pain ratings did not differ between conditions in patients with comorbid depression (*t*_75_ = 0.62, *p* = 0.80, Cohen’s *d* = 0.06 for baseline vs recovery; *t*_75_ = 1.50, *p* = 0.56, Cohen’s *d* = 0.11 for counter-irritation vs. recovery), see **Figure 2**. An exact Wilcoxon rank sum test indicated that the chosen intensity of the electrocutaneous stimulus was equally distributed in patients with and patients without comorbid depression (*Z* = -0.07, *p* = 0.94).

#### Effect of Baseline RMSSD Within Patients

When controlling for RMSSD, the effect of phase on pain ratings was not significant (main effect of phase: *F*_2,71_ = 0.15, *p* = 0.86). RMSSD did not influence overall pain ratings during the counter-irritation paradigm (main effect of RMSSD: *F*_1,71_ = 1.96, *p* = 0.17), and there was no RMSSD × phase interaction effect (*F*_2,71_ = 0.08, *p* = 0.92). Therefore, follow-up analyses were not performed.

### Additional Analyses

Because the magnitude of the counter-irritation effect is influenced by the painfulness of the counter-irritation stimulus (Granot et al., 2008) and the test stimulus (Yarnitsky et al., 2015), by age (Larivière et al., 2007) and by mental stress levels (Nilsen et al., 2012), we tested if these variables could explain the lack of expected findings. Furthermore, we explored the influence of the chronic use of pain medication on the counter-irritation effect.

#### Effect of Counter-Irritation Stimulus Painfulness

**FIGURE 3:**
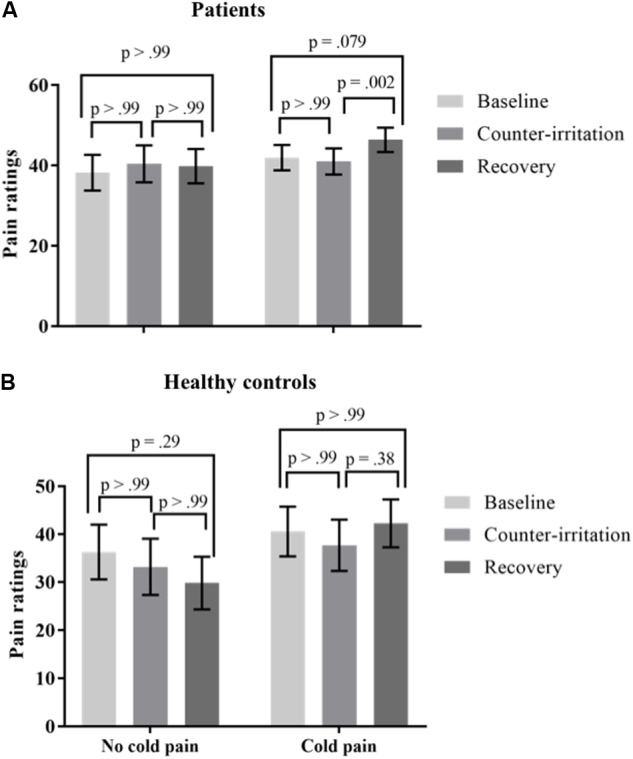
Least square mean pain ratings for electrocutaneous stimuli for **(A)** patients and **(B)** Healthy controls (HC) who did and did not experience the counter-irritation stimulus as painful [cold pressor arm wrap (CPAW) pain rating ≥20] in the baseline, counter-irritation and recovery phase. 23 patients and 14 HC found the CPAW not painful, 46 patients and 17 HC found the CPAW painful. *p*-values are corrected for multiple testing with the stepdown Bonferroni method. Vertical bars denote standard errors.

It has been stated that the painfulness of the counter-irritation stimulus is not related to EPM magnitude, as long as the counter-irritation stimulus is noxious (Granot et al., 2008). A pain score of at least 20/100 is recommended for the counter-irritation stimulus (Yarnitsky et al., 2015). Therefore, participants were divided in two “cold pain groups”: those that experienced pain caused by the CPAW (cold pain ratings ≥20; 46 patients, 17 controls) and those that did not (23 patients, 14 controls). Cold pain ratings were missing for 2 HC and 9 patients. An exact Wilcoxon rank sum test indicated that the distribution of the used intensity of electrocutaneous stimulation was equal in both cold pain groups (*Z* = 1.00, *p* = 0.32 for patients; *Z* = 0.53, *p* = 0.60 for controls). To investigate if the pain experienced by the CPAW (cold pain) influenced the results of the counter-irritation paradigm, we performed a mixed model analysis on the entire sample with phase, group (patient vs. HC) and cold pain group (cold pain vs. no cold pain) as fixed effects and electrocutaneous pain ratings as the dependent variable. The main effect of condition (*F*_2,97_ = 1.58, *p* = 0.21), the main effect of cold pain group (*F*_1,97_ = 1.39, *p* = 0.24) and the main effect of group (F*_1_*_,97_ = 1.04, *p* = 0.31) on electrocutaneous pain ratings were not significant. There was, however, a trend for a group × condition interaction effect (*F*_2,97_ = 2.89, *p* = 0.060) and a significant condition × cold pain group interaction effect (*F*_2,97_ = 5.85, *p* = 0.004). The three-way interaction was not significant. (*F*_3,97_ = 0.51, *p* = 0.67). Follow-up tests indicated that electrocutaneous pain ratings did not differ between the baseline and counter-irritation phase, regardless of group and cold pain group (*t*_97_ = -1.40, *p* > 0.99 for HC without cold pain; *t*_97_ = -1.45, *p* > 0.99 for HC with cold pain; *t*_97_ = 1.29, *p* > 0.99 for patients without cold pain; *t*_97_ = -0.79, *p* > 0.99 for patients with cold pain). However, patients that experienced cold pain gave higher pain ratings in the recovery phase than in the counter-irritation phase (*t*_97_ = 3.35, *p* = 0.002), while patients who did not experience cold pain and HC did not (*t*_97_ = -0.31, *p* > 0.99 for patients without cold pain; *t*_97_ = -1.37, *p* > 0.99 for HC without cold pain; *t*_97_ = 2.06, *p* = 0.38 for HC with cold pain), see **Figure 3**.

#### Effect of Test Stimulus Painfulness

**FIGURE 4:**
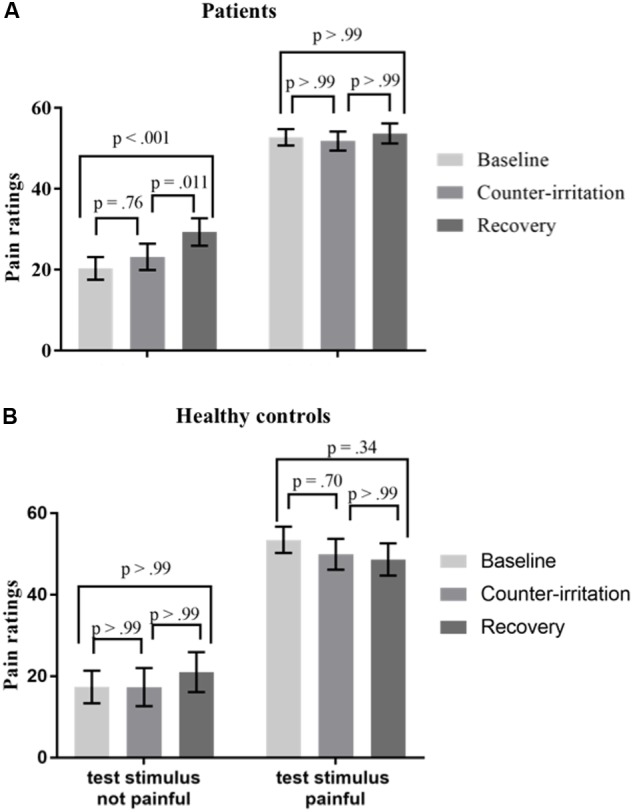
Least square mean pain ratings for electrocutaneous stimuli for **(A)** patients and **(B)** HC who did and did not experience the test stimulus as painful (average electrocutaneous pain rating in baseline ≥40) in the baseline, counter-irritation, and recovery phase. 27 patients and 13 HC found the test stimulus not painful, 51 patients and 20 HC found the test stimulus painful. *p*-values are corrected for multiple testing with the stepdown Bonferroni method. Vertical bars denote standard errors.

To investigate if the pain experienced by the test stimulus (electrocutaneous stimuli) influenced the results of the counter-irritation paradigm, we performed a mixed model analysis on the entire sample with phase, group and test stimulus pain as fixed effects and electrocutaneous pain ratings as the dependent variable. It is recommended that the test stimulus has a subjective painfulness of around 40/100 (Yarnitsky et al., 2015). Participants were thus divided in two groups (test pain group): those that experienced pain caused by the electrocutaneous stimuli (average ratings in the baseline phase ≥40; 42 patients, 20 controls) and those that did not (36 patients, 13 controls). An exact Wilcoxon rank sum test indicated that the distribution of the used electrocutaneous stimulus intensity was equal in both groups (*Z* = 0.95, *p* = 0.34 for patients; *Z* = 1.18, *p* = 0.24 for HC). To investigate if the pain intensity elicited by the test stimulus influenced the results of the counter-irritation paradigm, we performed a mixed model analysis on the entire sample with phase, group (patient vs. HC) and test pain group (test stimulus painful vs. test stimulus not painful) as fixed effects and electrocutaneous pain ratings as the dependent variable. There were no significant main effects of condition (*F*_2,108_ = 1.94, *p* = 0.15) or group (*F*_2,108_ = 0.54, *p* = 0.46) on electrocutaneous pain ratings. There were significant group × condition (*F*_2,108_ = 3.14, *p* = 0.047) and condition × test pain group (*F*_2,108_ = 3.28, *p* = 0.042) interaction effects, but no significant three-way interaction effect (*F*_3,108_ = 0.54, *p* = 0.66). Follow-up tests indicated that electrocutaneous pain ratings did not differ between the baseline and counter-irritation phase, regardless of group and test pain group (*t*_108_ = -0.54, *p* < 0.99 for HC who did not experience the electrocutaneous stimuli as painful; *t*_108_ = -1.59, *p* = 0.80 for HC who did experience the electrocutaneous stimuli as painful; *t*_108_ = 2.13, *p* = 0.36 for patients who did not experience the electrocutaneous stimuli as painful; *t*_108_ = -1.73, *p* = 0.69 for patients who did experience the electrocutaneous stimuli as painful). However, patients who did *not* experience the test stimulus as painful gave higher pain ratings in the recovery phase than in the baseline phase (*t*_108_ = 3.70, *p* = 0.004), while patients who *did* experience the test stimulus as painful and HC did not (*t*_108_ = 0.62, *p* > 0.99 for patients who did experience the test stimulus as painful; *t*_108_ = -1.54, *p* = 0.80 for HC who did experience the test stimulus as painful; *t*_108_ = 0.20, *p* > 0.99 for HC who didn’t experience the test stimulus as painful), see **Figure 4**.

#### Effect of Age

It has been shown that EPM efficiency declines with age, and that EPM efficiency is already smaller in middle-aged (40–55) individuals compared to young (20–35) individuals (Larivière et al., 2007). Over 54% of our sample was older than 40. To investigate if the participants’ age influenced the results of the counter-irritation paradigm, we performed a mixed model analysis on the entire sample with phase, group and age (continuously) as fixed effects and electrocutaneous pain ratings as the dependent variable. There was no significant main effect of age on electrocutaneous pain ratings (*F*_2,108_ = 0.03, *p* = 0.86). There were no significant interaction effects (*F*_2,108_ = 1.46, *p* = 0.24 for the group × condition interaction effect; *F*_2,108_ = 1.89, *p* = 0.16 for the age × condition interaction effect; *F*_3,108_ = 0.95, *p* = 0.42 for the three-way interaction effect). Therefore, follow-up analyses were not performed.

#### Effect of State Negative Affect

It has been shown that EPM efficiency is reduced in conditions of mental stress (Nilsen et al., 2012). Since this study was part of a larger project and the participants had already been in a test session for 2 h before doing the counter-irritation paradigm, stress and fatigue might have played a role. To investigate if the participants’ stress levels influenced the results of the counter-irritation paradigm, we performed a mixed model analysis on the entire sample with phase, group (patients vs. HC) and state negative affect (continuously) as fixed effects and electrocutaneous pain ratings as the dependent variable. Participants’ levels of state negative affect, measured immediately before the start of the calibration procedure, were rather low (11.37 for HC and 13.76 for patients, while the theoretical range of the state PANAS is 10–50). There was no significant main effect of state negative affect on electrocutaneous pain ratings (*F*_1,108_ = 0.92, *p* = 0.34). There were no significant interaction effects (*F*_2,107_ = 1.28, *p* = 0.28 for the group^∗^condition interaction effect; *F*_2,107_ = 1.43, *p* = 0.25 for the state negative affect × condition interaction effect; *F*_3,107_ = 2.58, *p* = 0.058 for the three-way interaction effect). Therefore, follow-up analyses were not performed.

#### Effect of Medication Use Within Patients

EPM efficiency might be influenced by the chronic intake of medication. Since a large number of patients were taking analgesics and/or antidepressants, we investigated the effect of analgesic use and antidepressant use on the results of the counter-irritation paradigm within the patient sample alone. Antidepressant use did not influence overall pain ratings during the counter-irritation paradigm (main effect of antidepressants: *F*_1,74_ = 0.47, *p* = 0.49), and there was no analgesics × phase interaction effect (*F*_2,74_ = 1.08, *p* = 0.35). Therefore, follow-up analyses were not performed. Analgesic use did not influence overall pain ratings during the counter-irritation paradigm (main effect of analgesics: *F*_1,76_ = 0.12, *p* = 0.73), and there was no analgesics × phase interaction effect (*F*_2,76_ = 1.19, *p* = 0.31). Therefore, follow-up analyses were not performed. Since there is evidence that the chronic use of opioids specifically can influence the perception of experimentally induced pain (Pud et al., 2006), we also investigated the effect of opioid use on the results of the counter-irritation paradigm within the patient sample alone. Opioid use did not influence overall pain ratings during the counter-irritation paradigm (main effect of opioids: *F*_1,74_ = 0.19, *p* = 0.66), and there was no opioids × phase interaction effect (*F*_2,74_ = 1.63, *p* = 0.20). Therefore, follow-up analyses were not performed.

## Discussion

The goal of the current study was to investigate moderators of EPM efficiency in a sample of patients with fibromyalgia and/or CFS, with a focus on NA, depression and resting HRV, quantified by RMSSD. For this purpose, 78 patients diagnosed with fibromyalgia and/or CFS and 33 age- and gender-matched HC completed a counter-irritation paradigm. Pain ratings elicited by noxious electrocutaneous stimulation were compared before (baseline), during (counter-irritation) and after (recovery) application of a CPAW. A larger reduction in pain ratings during counter-irritation (compared to pain ratings in the baseline phase) indicates more efficient EPM. In replication of previous studies, we expected that HC would have more efficient EPM than patients and that EPM efficiency within the patient group would be moderated by NA, the presence of a depression and by baseline RMSSD.

In contrast to our expectations, neither patients nor HC experienced the electrocutaneous stimuli as less painful in the counter-irritation compared to the baseline phase. Thus, we were not able to replicate the counter-irritation effect in our sample, making it impossible to draw valid conclusions on differences in EPM efficiency between patients and controls and on moderators of EPM efficiency within the patient group. It is known that the counter-irritation effect is influenced by and dependent on several methodological factors (Granot et al., 2008; Pud et al., 2009; Nir and Yarnitsky, 2015). Therefore, we performed additional analyses to investigate the effect of some of these factors on counter-irritation effect magnitude.

A first factor is the painfulness of the counter-irritation stimulus. Granot et al. (2008) proposed that while the level of pain intensity of the counter-irritation stimulus is not related to the magnitude of the counter-irritation effect, the counter-irritation stimulus must at least evoke mild pain to obtain a significant reduction in test stimulus painfulness. However, our results suggest that even in controls who did perceive the counter-irritation stimulus as painful, the counter-irritation effect was absent. Next, we investigated the influence of the test stimulus painfulness. It is suggested that for a significant reduction in test stimulus painfulness in response to the counter-irritation stimulus to appear, the test stimulus must at least be moderately painful. A pain intensity score of 40/100 is recommended in this regard (Yarnitsky et al., 2015). The intensity of our test stimulus was individually calibrated during a procedure in which participants received electrocutaneous stimuli of increasing intensity. Although all participants were encouraged to pick a stimulus intensity that was painful and required some effort to tolerate, a substantial number of participants (27 patients and 13 HC) rated the pain intensity of the test stimulus as lower than 40/100 in the baseline phase. However, when distinguishing the participants who did and did not find the test stimulus painful, no evidence of substantial pain reduction in the test stimulus due to counter-irritation was found in patients or controls. Because EPM magnitude is also influenced by age (Larivière et al., 2007) and mental stress (Nilsen et al., 2012), we investigated the relationship between the counter-irritation effect and age and state negative affect, but these factors did not influence our results. Because no significant counter-irritation effect could be elicited with the current paradigm in this sample, no valid conclusions can be drawn with regards to the moderating role of NA, depression and baseline RMSSD on the counter-irritation effect within the patient group.

Some specific findings deserve further attention. While there was no difference in pain ratings for the electrocutaneous stimuli between the baseline phase and the counter-irritation phase, patients overall rated the electrocutaneous stimuli in the recovery phase as more painful than the electrocutaneous stimuli in the counter-irritation phase and the baseline phase. These results suggest some sort of “rebound”-effect in patients, where the removal of the counter-irritation stimulus causes a reinstatement of pain elicited by the electrocutaneous stimulus. Only patients without comorbid depression showed this reinstatement of pain. Although such a reinstatement is often found in studies using similar paradigms, it is generally found in HC following successful pain reduction in the counter-irritation phase (Cormier et al., 2013; Piché et al., 2013), while in our study, this effect was only present in patients and after unsuccessful counter-irritation. Moreover, our additional analyses showed that this effect was driven by patients who perceived the cold pain stimulus as painful and by patients who perceived the test stimulus in the baseline phase as not painful (see **Figures 3**, **4**). Most counter-irritation paradigms and conditioned pain modulation paradigms compare pain ratings during or after counter-irritation with pain ratings before counter-irritation (Nir and Yarnitsky, 2015; Yarnitsky et al., 2015), with the expectation that pain ratings after counter-irritation will be either comparable to pain ratings during counter-irritation (continuance of counter-irritation effect throughout recovery; Piché et al., 2011; Ladouceur et al., 2012) or similar to pain ratings in the baseline phase (reinstatement of pain after counter-irritation; Cormier et al., 2013; Piché et al., 2013). Since in our study pain ratings in the recovery phase were higher than pain ratings in both baseline and counter-irritation phase, but only in patients and not in HC, this effect is difficult to interpret.

There are a number of limitations to our study that might explain the absence of an overall counter-irritation effect in this study. First, the paradigm used was adapted from Piché et al. (2011). In the original paradigm, every phase consisted of ten electrocutaneous stimuli, and pain ratings were given for the phase as a whole. We opted for only five compared to ten stimuli in each phase because the study was part of a larger project, and we did not want to overburden our participants. Furthermore, in our study, pain ratings were given after each stimulus compared to after each phase in the original paradigm. Short phase durations might not have allowed for stabilization of the effect, and the different timing of the pain ratings might have caused a difference in attentional focus. Second, our sample consisted primarily of women, which is a correct representation of the fibromyalgia and CFS population (Branco et al., 2010; Dantoft et al., 2017). It has been demonstrated that EPM is reduced in women (Pud et al., 2009), with some studies reporting that the DNIC-effect can only be elicited in men (Staud et al., 2003). Because of the small number of men participating in our study, we could not statistically control for this. Third, this study was part of a larger project investigating symptom perception processes and as a consequence the participants had already participated in the test session for 2 h before completing the counter-irritation paradigm. Although the test session did not include any other tasks involving experimental pain induction, it is very likely that fatigue might have influenced the results reported here. Although there are, to our knowledge, no studies investigating the role of experimentally induced fatigue on pain perception, it has been shown that sleep deprivation increases pain sensitivity (Lautenbacher et al., 2006) and that mental fatigue as a consequence of high levels of stimulation increases pain ratings (Marek et al., 1988). Finally, the instructions that are given to the participants influence the success of the counter-irritation paradigm. For instance, it has been shown that there is no modulation of test stimulus pain ratings by counter-irritation in participants who expect a hyperalgesic effect of the counter-irritation stimulus on test stimulus pain (Cormier et al., 2013). We did not measure participants’ expectations. However, we did use the following standardized instructions after calibration: “During this task, you will receive some stimuli on your ankle. This stimulus might vary in intensity throughout the task, but will never be higher than the intensity we agreed upon. After every stimulus, you will be asked to rate that stimulus on a rating scale. At some point in time, I will come in and apply an extra stimulus to your arm, a cold stimulus. When you are asked for pain ratings regarding the stimulus on your ankle, it’s important you only evaluate the pain elicited by the stimulus on your ankle.” Furthermore, the counter-irritation effect is reduced in participants who pay attention to the test stimulus compared to the counter-irritation stimulus (Ladouceur et al., 2012). It is likely that in our study, participants were very focused on the test stimulus, since they were prompted for electrocutaneous pain ratings after every stimulus.

The aim of this study was to investigate individual differences in EPM in a sample of patients with fibromyalgia and/or CFS by means of a counter-irritation paradigm. We focused on NA, comorbid depression, and HRV in rest as possible moderators within the patients group. However, we did not manage to successfully create a counter-irritation effect in patients or HC. Therefore, no valid conclusions on the moderating effect of NA, depression and RMSSD within the patient group can be drawn from these results. Although the DNIC-mechanism is a well-described phenomenon and the counter-irritation paradigm is widely used as a measure of EPM-efficiency, our results suggests that the counter-irritation effect is not always replicable. This brings about the question if the current knowledge on EPM and its significance for chronic pain conditions might be narrowed by positive publication bias. In any case, the methodologies between different studies employing one form of the counter-irritation paradigm vary widely, and success/failure of the paradigm might depend on the modality, intensity and timing of the used stimuli, and on which outcome statistic and data-analysis is chosen for the interpretation of the results (Granot et al., 2008; Pud et al., 2009; Nir and Yarnitsky, 2015). Currently, studies describing differences in EPM efficiency between HC and FSS patients are not always consistent, with results ranging from an absolute absence of DNIC in patients to no difference between patients and controls (Lewis et al., 2012). This large variety in possible methodologies and outcome variables of the counter-irritation paradigm constitutes a problem for the reliability of knowledge on the importance of EPM in chronic pain conditions. Furthermore, assurance that a particular form of the paradigm is a valid assessment of EPM efficiency is essential when it is used as a clinical tool on an individual level, for instance with the purpose of identifying individuals at risk for developing chronic pain or in order to inform the appropriate treatment strategy. Recently, a number of recommendations on the methodological choices in counter-irritation paradigms have been published in order to increase uniformity in methodology (Yarnitsky et al., 2015). Researchers aiming to investigate EPM efficiency in clinical populations are well-advised to follow these recommendations, in order to increase comparability between studies and establish to what extent EPM is of etiological, clinical, and therapeutical relevance for chronic pain.

## Author Contributions

MVDH was responsible for the data collection and data analysis. All authors contributed to the conception or design of the study, to the interpretation of the data, and to the drafting or revising of the manuscript. All authors have approved the final version of the manuscript. All authors agreed to be accountable for all aspects of the work.

## Conflict of Interest Statement

The authors declare that the research was conducted in the absence of any commercial or financial relationships that could be construed as a potential conflict of interest.

## Abbreviations

CFS: chronic fatigue syndrome
CPAW: cold pressor arm wrap
DNIC: diffuse noxious inhibitory controls
EPM: endogenous pain modulation
FSS: functional somatic syndromes
HC: healthy controls
HRV: heart rate variability
NA: negative affectivity
RMSSD: root mean square of the successive differences of R–R intervals

## References

[B1] Bourke J. H. Langford R. M. White P. D. (2015). The common link between functional somatic syndromes may be central sensitisation. *J. Psychosom. Res.* 78 228–236. 10.1016/j.jpsychores.2015.01.003 25598410

[B2] Bourne S. Machado A. G. Nagel S. J. (2014). Basic anatomy and physiology of pain pathways. *Neurosurg. Clin. N. Am.* 25 629–638. 10.1016/j.nec.2014.06.001 25240653

[B3] Branco J. C. Bannwarth B. Failde I. Carbonell J. A. Blotman F. Spaeth M. (2010). Prevalence of fibromyalgia: a survey in five European countries. *Semin. Arthritis Rheum.* 39 448–453. 10.1016/j.semarthrit.2008.12.003 19250656

[B4] Cormier S. Piché M. Rainville P. (2013). Expectations modulate heterotopic noxious counter-stimulation analgesia. *J. Pain* 14 114–125. 10.1016/j.jpain.2012.10.006 23260452

[B5] Cosci F. Pennato T. Bernini O. Berrocal C. (2011). Psychological well-being, negative affectivity, and functional impairment in fibromyalgia. *Psychother. Psychosom.* 80 256–258. 10.1159/000322031 21546785

[B6] Dantoft T. M. Ebstrup J. F. Linneberg A. Skovbjerg S. Madsen A. L. Mehlsen A. L. (2017). Cohort description: the Danish study of functional disorders. *Clin. Epidemiol.* 9 127–139. 10.2147/CLEP.S129335 28275316 PMC5333638

[B7] de Souza J. B. Potvin S. Goffaux P. Charest J. Marchand S. (2009). The deficit of pain inhibition in fibromyalgia is more pronounced in patients with comorbid depressive symptoms. *Clin. J. Pain* 25 123–127. 10.1097/AJP.0b013e318183cfa4 19333157

[B8] Edwards R. R. (2005). Individual differences in endogenous pain modulation as a risk factor for chronic pain. *Neurology* 65 437–443. 10.1212/01.wnl.0000171862.17301.84 16087910

[B9] Fukuda K. Straus S. Hickie I. Sharpe M. Dobbins J. Komaroff A. (1994). The chronic fatigue syndrome: a comprehensive approach to its definition and study. *Ann. Intern. Med.* 121 953–959. 10.7326/0003-4819-121-12-199412150-00009 7978722

[B10] Granot M. Weissman-Fogel I. Crispel Y. Pud D. Granovsky Y. Sprecher E. (2008). Determinants of endogenous analgesia magnitude in a diffuse noxious inhibitory control (DNIC) paradigm: do conditioning stimulus painfulness, gender and personality variables matter? *Pain* 136 142–149. 10.1016/j.pain.2007.06.029 17720319

[B11] Harkins S. W. Price D. D. Braith J. (1989). Effects of extraversion and neuroticism on experimental pain, clinical pain, and illness behavior. *Pain* 36 209–218. 10.1016/0304-3959(89)90025-0 2919101

[B12] Henningsen P. Zimmerman T. Sattel H. (2003). Medically unexplained physical symptoms, anxiety, and depression: a meta-analytic review. *Psychosom. Med.* 65 528–533. 10.1097/01.PSY.0000075977.90337.E7 12883101

[B13] Jacobs B. L. Azmitia E. C. (1992). Structure and function of the brain serotonin system. *Physiol. Rev.* 72 165–229. 10.1152/physrev.1992.72.1.165 1731370

[B14] Jensen K. B. Kosek E. Petzke F. Carville S. Fransson P. Marcus H. (2009). Evidence of dysfunctional pain inhibition in Fibromyalgia reflected in rACC during provoked pain. *Pain* 144 95–100. 10.1016/j.pain.2009.03.018 19410366

[B15] Julien N. Goffaux P. Arsenault P. Marchand S. (2005). Widespread pain in fibromyalgia is related to a deficit of endogenous pain inhibition. *Pain* 114 295–302. 10.1016/j.pain.2004.12.032 15733656

[B16] Kaufmann T. Sütterlin S. Schulz S. M. Vögele C. (2011). ARTiiFACT: a tool for heart rate artifact processing and heart rate variability analysis. *Behav. Res. Methods* 43 1161–1170. 10.3758/s13428-011-0107-7 21573720

[B17] Koenig J. Jarczok M. N. Ellis R. J. Hillecke T. K. Thayer J. F. (2014). Heart rate variability and experimentally induced pain in healthy adults: a systematic review. *Eur. J. Pain* 18 301–314. 10.1002/j.1532-2149.2013.00379.x 23922336

[B18] Lacourt T. E. Houtveen J. H. Veldhuijzen Van Zanten J. J. C. S. Bosch J. A. Drayson M. T. Van Doornen L. J. P. (2015). Negative affectivity predicts decreased pain tolerance during low-grade inflammation in healthy women. *Brain Behav. Immun.* 44 32–36. 10.1016/j.bbi.2014.10.003 25451608

[B19] Ladouceur A. Tessier J. Provencher B. Rainville P. Piché M. (2012). Top-down attentional modulation of analgesia induced by heterotopic noxious counterstimulation. *Pain* 153 1755–1762. 10.1016/j.pain.2012.05.019 22717100

[B20] Larivière M. Goffaux P. Marchand S. Julien N. (2007). Changes in pain perception and descending inhibitory controls start at middle age in healthy adults. *Clin. J. Pain* 23 506–510. 10.1097/AJP.0b013e31806a23e8 17575490

[B21] Lautenbacher S. Kundermann B. Krieg J. C. (2006). Sleep deprivation and pain perception. *Sleep Med. Rev.* 10 357–369. 10.1016/j.smrv.2005.08.001 16386930

[B22] Lautenbacher S. Rollman G. (1997). Possible deficiencies of pain modulation in fibromyalgia. *Clin. J. Pain* 13 189–196. 10.1097/00002508-199709000-00003 9303250

[B23] Le Bars D. Dickenson A. H. Besson J.-M. (1979). Diffuse noxious inhibitory controls (DNIC). I. Effects on dorsal horn convergent neurones in the rat. *Pain* 6 283–304. 10.1016/0304-3959(79)90049-6 460935

[B24] Lewis G. N. Rice D. A. McNair P. J. (2012). Conditioned pain modulation in populations with chronic pain: a systematic review and meta-analysis. *J. Pain* 13 936–944. 10.1016/j.jpain.2012.07.005 22981090

[B25] Marek T. Noworol C. Karwowski W. (1988). Mental fatigue at work and pain perception. *Work Stress* 2 133–137. 10.1080/02678378808259157

[B26] Mazurak N. Seredyuk N. Sauer H. Teufel M. Enck P. (2012). Heart rate variability in the irritable bowel syndrome: a review of the literature. *Neurogastroenterol. Motil.* 24 206–216. 10.1111/j.1365-2982.2011.01866.x 22256893

[B27] Meeus M. Goubert D. De Backer F. Struyf F. Hermans L. Coppieters I. (2013). Heart rate variability in patients with fibromyalgia and patients with chronic fatigue syndrome: a systematic review. *Semin. Arthritis Rheum.* 43 279–287. 10.1016/j.semarthrit.2013.03.004 23838093

[B28] Meeus M. Nijs J. Van de Wauwer N. Toeback L. Truijen S. (2008). Diffuse noxious inhibitory control is delayed in chronic fatigue syndrome: an experimental study. *Pain* 139 439–448. 10.1016/j.pain.2008.05.018 18617327

[B29] Nahman-Averbuch H. Dayan L. Sprecher E. Hochberg U. Brill S. Yarnitsky D. (2015). Sex differences in the relationships between parasympathetic activity and pain modulation. *Physiol. Behav.* 154 40–48. 10.1016/j.physbeh.2015.11.004 26556539

[B30] Nahman-Averbuch H. Nir R. Sprecher E. Yarnitsky D. (2016). Psychological factors and conditioned pain modulation. *Clin. J. Pain* 32 541–554. 10.1097/AJP.0000000000000296 26340657

[B31] Naughton M. Mulrooney J. B. Leonard B. E. (2000). A review of the role of serotonin receptors in psychiatric disorders. *Hum. Psychopharmacol.* 15 397–415. 10.1002/1099-1077(200008)15:6<397::AID-HUP212>3.0.CO;2-L 12404302

[B32] Nilsen K. B. Christiansen S. E. Holmen L. B. Sand T. (2012). The effect of a mental stressor on conditioned pain modulation in healthy subjects. *Scand. J. Pain* 3 142–148. 10.1016/j.sjpain.2012.04.005 29913861

[B33] Nir R. R. Yarnitsky D. (2015). Conditioned pain modulation. *Curr. Opin. Support. Palliat. Care* 9 131–137. 10.1097/SPC.0000000000000126 25699686

[B34] Overbeek T. Schruers K. Griez E. (1999). *MINI International Neuropsychiatric Interview, Dutch Version 5.0.0 (DSM-IV).* Maastricht: University of Maastricht.

[B35] Piché M. Bouin M. Arsenault M. Poitras P. Rainville P. (2011). Decreased pain inhibition in irritable bowel syndrome depends on altered descending modulation and higher-order brain processes. *Neuroscience* 195 166–175. 10.1016/j.neuroscience.2011.08.040 21889972

[B36] Piché M. Chen J. Roy M. Poitras P. Bouin M. Rainville P. (2013). Thicker posterior insula is associated with disease duration in women with Irritable Bowel Syndrome (IBS) whereas thicker orbitofrontal cortex predicts reduced pain inhibition in both IBS patients and controls. *J. Pain* 14 1217–1226. 10.1016/j.jpain.2013.05.009 23871603

[B37] Porcelli A. J. (2014). An alternative to the traditional cold pressor test: the cold pressor arm wrap. *J. Vis. Exp.* 83:e50849. 10.3791/50849 24457998 PMC4089407

[B38] Potvin S. Marchand S. (2016). Pain facilitation and pain inhibition during conditioned pain modulation in fibromyalgia and in healthy controls. *Pain* 157 1704–1710. 10.1097/j.pain.0000000000000573 27045524

[B39] Pud D. Cohen D. Lawental E. Eisenberg E. (2006). Opioids and abnormal pain perception: new evidence from a study of chronic opioid addicts and healthy subjects. *Drug Alcohol Depend.* 82 218–223. 10.1016/j.drugalcdep.2005.09.007 16229972

[B40] Pud D. Granovsky Y. Yarnitsky D. (2009). The methodology of experimentally induced diffuse noxious inhibitory control (DNIC)-like effect in humans. *Pain* 144 16–19. 10.1016/j.pain.2009.02.015 19359095

[B41] Sheehan D. V. Lecrubier Y. Sheehan K. H. Amorim P. Janavs J. Weiller E. (1998). The Mini-International Neuropsychiatric Interview (M.I.N.I.): the development and validation of a structured diagnostic psychiatric interview for DSM-IV and ICD-10. *J. Clin. Psychiatry* 59 22–33. 9881538

[B42] Smith T. W. Mackenzie J. (2006). Personality and risk of physical illness. *Annu. Rev. Clin. Psychol.* 2 435–467. 10.1146/annurev.clinpsy.2.022305.095257 17716078

[B43] Staud R. (2012). Abnormal endogenous pain modulation is a shared characteristic of many chronic pain conditions. *Expert Rev. Neurother.* 12 577–585. 10.1586/ern.12.41 22550986 PMC3373184

[B44] Staud R. Robinson M. E. Vierck C. J. Price D. D. (2003). Diffuse noxious inhibitory controls (DNIC) attenuate temporal summation of second pain in normal males but not in normal females or fibromyalgia patients. *Pain* 101 167–174. 10.1016/S0304-3959(02)00325-1 12507711

[B45] Thayer J. F. Ahs F. Fredrikson M. Sollers J. J. III Wager T. D. (2012). A meta-analysis of heart rate variability and neuroimaging studies: implications for heart rate variability as a marker of stress and health. *Neurosci. Biobehav. Rev.* 36 747–756. 10.1016/j.neubiorev.2011.11.009 22178086

[B46] Treister R. Pud D. Ebstein R. P. Laiba E. Raz Y. Gershon E. (2011). Association between polymorphisms in serotonin and dopamine-related genes and endogenous pain modulation. *J. Pain* 12 875–883. 10.1016/j.jpain.2011.02.348 21719351

[B47] Tsao J. C. I. Seidman L. C. Evans S. Lung K. C. Zeltzer L. K. Naliboff B. D. (2013). Conditioned pain modulation in children and adolescents: effects of sex and age. *J. Pain* 14 558–567. 10.1016/j.jpain.2013.01.010 23541066 PMC3672325

[B48] Van Den Houte M. Bogaerts K. Van Diest I. De Bie J. Persoons P. Van Oudenhove L. (2017a). Inducing somatic symptoms in functional syndrome patients: effects of manipulating state negative effect. *Psychosom. Med.* 79 1000–1007. 10.1097/PSY.0000000000000527 28914723

[B49] Van Den Houte M. Bogaerts K. Van Diest I. De Bie J. Persoons P. Van Oudenhove L. (2018). Perception of induced dyspnea in fibromyalgia and chronic fatigue syndrome. *J. Psychosom. Res.* 106 49–55. 10.1016/j.jpsychores.2018.01.007 29455899

[B50] Van Den Houte M. Van Oudenhove L. Bogaerts K. Van Diest I. Van den Bergh O. (2017b). Endogenous pain modulation: association with resting heart rate variability and negative affectivity. *Pain Med.* 10.1093/pm/pnx165 [Epub ahead of print]. 29016885

[B51] Van Diest I. De Peuter S. Eertmans A. Bogaerts K. Victoir A. Van den Bergh O. (2005). Negative affectivity and enhanced symptom reports: differentiating between symptoms in men and women. *Soc. Sci. Med.* 61 1835–1845. 10.1016/j.socscimed.2005.03.031 16029779

[B52] Van Houdenhove B. Luyten P. (2008). Customizing treatment of chronic fatigue syndrome and fibromyalgia: the role of perpetuating factors. *Psychosomatics* 49 470–477. 10.1176/appi.psy.49.6.470 19122123

[B53] Watson D. Clark L. (1984). Negative affectivity: the disposition to experience aversive emotional states. *Psychol. Bull.* 96 465–490. 10.1037/0033-2909.96.3.465 6393179

[B54] Watson D. Clark L. A. Tellegen A. (1988). Development and validation of brief measures of positive and negative affect: the PANAS scales. *J. Pers. Soc. Psychol.* 54 1063–1070. 10.1037/0022-3514.54.6.1063 3397865

[B55] Wilder-Smith C. H. (2011). The balancing act: endogenous modulation of pain in functional gastrointestinal disorders. *Gut* 60 1589–1599. 10.1136/gutjnl-2011-300253 21768212

[B56] Wilder-Smith O. H. Schreyer T. Scheffer G. J. (2010). Patients with chronic pain after abdominal surgery show less preoperative endogenous pain inhibition and more postoperative hyperalgesia: a pilot study. *J. Pain Palliat. Care Pharmacother.* 24 119–128. 10.3109/15360281003706069 20504133

[B57] Wolfe F. Clauw D. J. Fitzcharles M.-A. Goldenberg D. L. Katz R. S. Mease P. (2010). The American college of rheumatology preliminary diagnostic criteria for fibromyalgia and measurement of symptom severity. *Arthritis Care Res.* 62 600–610. 10.1002/acr.20140 20461783

[B58] Yarnitsky D. (2015). Role of endogenous pain modulation in chronic pain mechanisms and treatment. *Pain* 156 24–31. 10.1097/01.j.pain.0000460343.46847.58 25789433

[B59] Yarnitsky D. Bouhassira D. Drewes A. M. Fillingim R. B. Granot M. Hansson P. (2015). Recommendations on practice of conditioned pain modulation (CPM) testing. *Eur. J. Pain* 19 805–806. 10.1002/ejp.605 25330039

[B60] Yarnitsky D. Crispel Y. Eisenberg E. Granovsky Y. Ben-Nun A. Sprecher E. (2008). Prediction of chronic post-operative pain: pre-operative DNIC testing identifies patients at risk. *Pain* 138 22–28. 10.1016/j.pain.2007.10.033 18079062

[B61] Zinbarg R. E. Mineka S. Bobova L. Craske M. G. Vrshek-Schallhorn S. Griffith J. W. (2016). Testing a hierarchical model of neuroticism and its cognitive facets: latent structure and prospective prediction of first onsets of anxiety and unipolar mood disorders during 3 years in late adolescence. *Clin. Psychol. Sci.* 4 805–824. 10.1177/2167702615618162

